# An AI-Driven Multimodal Sensing Framework Integrating UAV Imagery and Environmental Sensors for Intelligent Farmland Monitoring

**DOI:** 10.3390/s26113456

**Published:** 2026-05-30

**Authors:** Liangyu Li, Yiwei Song, Yintianrun Zhang, Peijiang Guo, Xi Wang, Zhenlin Ma, Shuo Yan

**Affiliations:** 1 China Agricultural University, Beijing 100083, China; 2 College of Plant Sciences, Xizang Agricultural and Animal Husbandry University, Linzhi 860000, China; 3 National School of Development, Peking University, Beijing 100871, China; 4 School of Automation Science and Electrical Engineering, Beihang University, Beijing 100191, China; 5 State Key Laboratory of Agricultural and Forestry Biosecurity, MARA Key Lab of Surveillance and Management for Plant Quarantine Pests, Beijing 100193, China

**Keywords:** multimodal agricultural sensing, AI-driven farmland monitoring, multimodal sensor fusion, environmental sensor data integration, precision agriculture decision support, agricultural E-commerce integration

## Abstract

The utilization of multi-source sensing data to achieve intelligent perception and refined management of farmland has become a vital research direction in modern agriculture. However, traditional inspection approaches based solely on visual information are highly susceptible to illumination variations, occlusion, and background interference, which makes stable pest detection and accurate crop growth assessment difficult to achieve. To address these problems, we propose a multimodal target perception network for intelligent farmland inspection. By integrating UAV imagery, ground environmental sensor data, and spatial location information, joint perception of farmland pests, diseases, and crop growth status is achieved. In the proposed framework, cross-modal alignment and collaborative encoding mechanisms, a multi-scale target perception structure, and a dynamic multimodal fusion strategy are introduced to collaboratively model information within a unified semantic space. Experimental results on a constructed multimodal farmland dataset demonstrate that the proposed method achieved 87.53% Precision and 89.16% mAP in the pest and disease detection task, and 88.04% Accuracy in the crop growth assessment task, significantly outperforming several mainstream visual detection models and multimodal fusion approaches. The results indicate that this intelligent perception framework can significantly improve the robustness of farmland inspection systems, providing an effective technical pathway for AI-driven precision agriculture decision-making. This technology breaks the barrier between production-side sensing data and e-commerce demand, providing a practical technical solution for agricultural production-marketing synergy, quality premium realization and digital rural revitalization.

## 1. Introduction

With the rapid development of smart agriculture and precision agriculture [1], farmland management is shifting from experience-based practices toward data-driven and intelligent decision-making [2]. Efficient and fine-grained farmland monitoring has become essential for ensuring stable agricultural production and improving resource utilization. Among related tasks, pest and disease monitoring and crop growth assessment are particularly important, as they directly affect crop yield, quality, risk warning, and precise intervention [3,4]. However, traditional manual inspection is inefficient, subjective, and difficult to apply to large-scale farmland. Therefore, automated inspection based on unmanned aerial vehicles (UAVs), ground sensors, and artificial intelligence has attracted increasing attention [5]. Concurrently, the rapid digitalization of agricultural markets has created new economic imperatives for intelligent field monitoring. The global precision agriculture market was valued at approximately USD 10.5 billion in 2024 and is projected to exceed USD 27 billion by 2033, reflecting an annual growth rate of more than 9% [6]. Empirical evidence further shows that precision agriculture adoption can increase farm-level return on investment and net profit while reducing pesticide use [7]. Meanwhile, pests and diseases continue to cause substantial yield losses in major food crops, including wheat and maize, imposing persistent economic pressure on global food supply chains [8]. These losses are closely related to delayed detection, insufficient intervention, and the lack of standardized quality data for market sorting and supply chain management. With the rapid growth of agricultural e-commerce platforms in China, reliable and verifiable crop quality information has become an increasingly important commercial asset. The joint adoption of digital sales channels and product quality certification has been shown to improve market access and profitability for agricultural producers [9]. Therefore, AI-driven inspection frameworks are expected to provide not only accurate agronomic monitoring results but also interpretable quality signals for market-oriented precision agriculture.

In recent years, vision-based farmland monitoring has become a mainstream research direction with the development of computer vision and deep learning [10]. Existing studies commonly use UAV or ground-camera images and apply convolutional neural networks (CNNs) or vision transformers for pest and disease recognition, crop growth estimation, and object detection [11]. Although these methods can achieve promising results in controlled or simple scenarios, they still rely mainly on a single visual modality and therefore face limitations in real agricultural environments [12]. In practice, weather changes, illumination variation, crop occlusion, flight-altitude differences, and background interference can significantly affect image quality and visual feature stability [13]. When visual information is degraded by noise or distortion, models may fail to accurately capture crop conditions, leading to unstable or inaccurate predictions [14]. Thus, the robustness and generalization ability of single-vision approaches remain insufficient for large-scale farmland inspection [15]. In addition to visual information, spatial and environmental factors are also important for characterizing crop growth and disease occurrence. Soil conditions, local microclimates, temperature, humidity, and soil moisture may all influence crop development and pest or disease outbreaks [16,17,18]. However, such information is often ignored or simply concatenated with visual features in existing studies [19]. This shallow fusion strategy lacks effective cross-modal collaboration and cannot fully exploit the complementary value of multi-source information [20].

To overcome these limitations, recent studies have begun to explore multimodal perception methods for agricultural monitoring [21]. Some works combine remote sensing imagery with environmental sensor data to improve crop state recognition [22], while others integrate visual and meteorological information for pest and disease risk prediction. Nevertheless, several challenges remain. First, different modalities often have significant distribution discrepancies, making effective semantic alignment difficult [23]. Second, farmland targets such as disease spots, pests, and crop canopy regions exhibit large scale variations [24]. Third, the reliability and importance of each modality may change under different environmental conditions, whereas many existing methods still adopt fixed-weight fusion strategies [25]. Therefore, more adaptive and systematic multimodal modeling strategies are needed. Related cross-modal learning studies have provided useful references for this problem. Hu et al. [26] proposed a transformer-based remote sensing image–text retrieval framework that improves retrieval performance through global–local information alignment. Chen et al. [27] introduced a scale-aware adaptive refinement and cross-modal interaction network for remote sensing audio–visual retrieval. Chen et al. [28] further improved cross-modal semantic alignment using global–local fusion guidance and bidirectional fine-grained guidance. These studies demonstrate the potential of cross-modal alignment and interaction mechanisms for improving representation robustness, offering useful inspiration for intelligent agricultural inspection.

To this end, this study proposes a synergetic multimodal target perception framework for intelligent farmland inspection. The framework integrates UAV imagery, ground environmental sensor data, and spatial location information to jointly perceive crop pests, diseases, and growth status. Specifically, a cross-modal alignment module is introduced to reduce distribution gaps between heterogeneous data sources, a multi-scale target modeling structure is designed to enhance the representation of farmland targets at different spatial scales, and a dynamic fusion mechanism is developed to adaptively integrate multi-source information according to data reliability. By combining these components, the proposed framework improves the stability of visual perception and enhances multimodal collaboration in complex agricultural scenarios.

The main contributions of this study are summarized as follows.

1. We introduce and open-source a highly structured, spatio-temporally aligned multimodal farmland inspection dataset. Comprising thousands of expertly annotated UAV images, continuous microclimatic sensor logs, and precise GPS coordinates, this publicly released dataset provides a reproducible benchmark to support the research community in exploring the complex interactions between visual crop phenotypes and localized environmental conditions.
2. We design a synergetic target perception framework that fundamentally moves beyond naive multimodal feature aggregation. By introducing a cross-modal alignment module and a dynamic reliability gating mechanism, the architecture explicitly models the non-linear correlations between high-dimensional visual symptoms and sparse microclimatic triggers, offering a robust architectural solution to the chronic instability of vision-only sensing under severe agricultural background interference.
3. Through comprehensive evaluation, we establish a new multi-source sensing paradigm that reveals the hierarchical role of heterogeneous data in precision agriculture. Rather than merely reporting performance metrics as an outcome, our empirical findings scientifically validate that while multi-scale visual extraction provides baseline morphological localization, the dynamic integration of environmental priors acts as the foundational pillar for overall system robustness, successfully bridging the gap between controlled algorithmic testing and practical field deployment.

## 2. Related Work

### 2.1. Vision-Based Farmland Inspection and Pest and Disease Recognition

Vision-based farmland inspection has long been an important research direction in smart agriculture [29]. Early methods mainly relied on handcrafted color, texture, or shape features combined with traditional classifiers, such as support vector machines (SVMs) and random forests, for crop disease classification [30]. However, the limited representation capability of handcrafted features often led to weak generalization in complex farmland environments. With the development of deep learning, convolutional neural networks (CNNs) have been widely used for crop disease recognition, phenotypic analysis, and growth assessment [31]. Bari et al. [32] proposed a real-time rice leaf disease diagnosis method based on Faster R-CNN, achieving accurate recognition of diseases such as rice blast and brown spot by combining public datasets with field images. Subsequently, detection frameworks such as Faster R-CNN, the YOLO series, and RetinaNet have been applied to UAV-based farmland object detection, including disease spots, pest distribution, and weed targets [33]. Semantic segmentation methods have also been used to extract crop regions and analyze growth conditions [34]. Despite these advances, vision-based methods still face challenges in real agricultural environments [35]. UAV images often contain both large-scale farmland structures and small disease spots, requiring strong multi-scale representation capability [36]. In addition, leaf overlap, dense crop growth, and weed interference may cause occlusion and missing visual features [37]. Illumination and weather variations, such as shadows, haze, and strong reflections, can further degrade image quality and reduce model stability [38]. Therefore, relying solely on visual information remains insufficient for robust farmland inspection in complex scenarios.

### 2.2. Application of Multi-Sensor Data in Smart Agriculture

To obtain a more comprehensive understanding of farmland ecosystems, environmental and soil sensors have been increasingly used in agricultural monitoring. With the development of agricultural Internet of Things (IoT), variables such as temperature, humidity, soil moisture, light intensity, and meteorological information can be continuously collected to support agricultural decision-making. Early studies mainly used sensor data for environmental monitoring, pest and disease risk prediction, and irrigation management [39]. These methods often relied on statistical models or traditional machine learning algorithms for environmental modeling and production management [40]. More recently, deep learning methods such as recurrent neural networks (RNNs) and long short-term memory networks (LSTMs) have been applied to sensor time-series data to predict crop growth conditions and agricultural risks [41]. Some studies have also integrated multi-source information into agricultural monitoring systems, for example by combining meteorological data with remote sensing imagery for crop yield prediction or pest and disease early warning [42]. These approaches have improved the predictive capability of agricultural monitoring systems. However, sensor data are still often used independently for trend analysis or decision support, rather than being deeply integrated with visual information [43]. In many systems, environmental variables are simply treated as auxiliary features or used in rule-based post-processing, lacking a unified end-to-end collaborative learning framework [44]. Moreover, visual data and sensor data differ substantially in structure and semantic representation, which makes effective heterogeneous data fusion challenging [45]. Therefore, multimodal models that jointly leverage visual phenotypic information and environmental context are needed to improve the accuracy and robustness of farmland inspection.

### 2.3. Multimodal Fusion and Cross-Modal Learning Methods

Multimodal learning aims to jointly model data from different modalities and exploit their complementary information [46]. It has been widely studied in computer vision, robotic perception, and autonomous driving [47]. Existing fusion strategies are commonly divided into early fusion, intermediate fusion, and late fusion. Early fusion directly concatenates features from different modalities, while late fusion combines prediction results from independently processed modalities. Although simple and widely used, these strategies often struggle to capture deep semantic relationships between modalities [48]. With the development of transformer architectures, attention mechanisms have been increasingly adopted for multimodal feature alignment and interaction. Cross-modal learning methods based on attention, shared embedding spaces, and contrastive learning have shown strong potential in establishing semantic correspondences between heterogeneous modalities [49,50]. In agriculture, multimodal approaches have also been explored to integrate remote sensing data, environmental information, and geographic data [51]. For example, Dilmurat et al. [52] combined UAV hyperspectral imagery with LiDAR data for maize yield prediction, while Meghraoui et al. [53] used multimodal remote sensing data for wheat yield estimation. These studies indicate that multimodal information can improve agricultural monitoring compared with single-modality methods. Nevertheless, several limitations remain. First, UAV imagery and ground sensor data often differ in sampling frequency and spatial distribution, making temporal and spatial alignment difficult [54]. Second, image data are high-dimensional spatial features, whereas environmental sensor data are typically low-dimensional time-series signals, which increases the difficulty of unified representation learning [55]. Third, many existing methods adopt fixed-weight fusion strategies and cannot dynamically adjust the contribution of each modality under different environmental conditions [56]. Therefore, a multimodal perception framework with effective cross-modal alignment, heterogeneous feature modeling, and adaptive fusion is needed for stable and efficient farmland inspection.

### 2.4. Agricultural E-Commerce, Product Traceability, and Digital Value Chain Integration

The convergence of intelligent agricultural sensing and digital commerce has become an important direction for modern agriculture. In China, rural e-commerce has reshaped agricultural supply chains by strengthening producer-to-consumer connections and reducing intermediary costs. Guo et al. [57] showed that digitally integrated agricultural supply chains exhibited stronger resilience during the COVID-19 period, while the Tudouec potato e-commerce platform in Inner Mongolia further demonstrated how digital platforms can reorganize material, information, and financial flows in agricultural supply chains [58]. Reliable product traceability is a key prerequisite for converting monitoring data into commercial value. Consumer studies indicate that buyers are willing to pay premiums for traceable agricultural products when supplementary quality assurance information is available [59]. Accordingly, blockchain-based traceability systems integrated with IoT sensor networks have been developed to provide tamper-resistant quality records across production and circulation processes [60]. The economic relevance of intelligent monitoring is also reflected in cross-border agricultural trade. Existing studies suggest that China’s cross-border e-commerce policies can promote agricultural export quality improvement, although quality verification infrastructure remains a constraint [61]. Liu et al. [9] further showed that the joint adoption of e-commerce and product quality certification generates higher market returns for agricultural producers. However, most existing studies still treat farm monitoring information as static labeling evidence, while dynamic, multimodal, and spatiotemporally aligned sensing data remain insufficiently explored for traceability, certification, and supply chain decision support. The proposed framework therefore provides structured field-level monitoring data to support both intelligent agronomic inspection and downstream digital value chain integration.

## 3. Materials and Method

### 3.1. Data Collection

**Figure 1:**
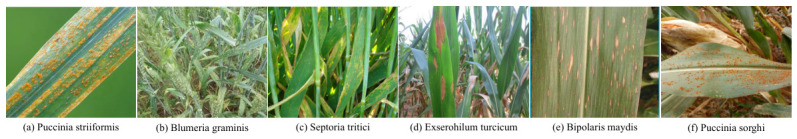
Schematic illustration of local representative crop disease dataset.

Data collection was conducted in a modern agricultural demonstration farmland located in Wuyuan County, Bayannur City, Inner Mongolia Autonomous Region, China. To further enhance the diversity of pest and disease phenotypic features, particularly for specific rare symptoms that exhibited low frequency during our 2024 field observation period, 800 additional images (representing approximately 12.5% of the visual dataset) were sourced from public agricultural research databases such as PlantVillage and IP102. These external images were strictly curated to ensure they closely matched the downward-looking perspective and spatial resolution characteristic of our UAV imagery. This region lies within the core agricultural zone of the Hetao Irrigation District and exhibits a typical temperate continental climate, characterized by distinct crop growth cycles and regionally representative pest and disease patterns, as shown in Figure 1 and Table 1. The experimental farmland covered an area of approximately 48 hectares and was primarily planted with wheat and maize. To ensure high-quality visual data, we utilized a DJI Matrice 300 RTK multirotor UAV equipped with a Zenmuse P1 full-frame camera (SZ DJI Technology Co., Ltd., Shenzhen, China) and a 35 mm fixed focal length lens. Considering the relatively flat topography and highly homogeneous planting structure of the demonstration area, the environmental variance across different plots is relatively contained. Data acquisition spanned the entire crop growing season, with periodic inspections conducted from May 2024 to September 2024. To enable multi-source perception of farmland environmental conditions and crop states, a multimodal farmland inspection dataset integrating UAV remote sensing imagery, ground environmental sensor data, and spatial location information was constructed. Crucially, all images, regardless of their source, underwent the same rigorous manual re-annotation protocol by our team of three agricultural experts using CVAT to ensure label and standard consistency. The 800 internet images are strictly isolated and utilized exclusively for the initial visual pre-training of the backbone network. The cross-modal alignment, the training of the synergetic fusion module, and all final performance evaluations are completely restricted to the 5620 field-captured images that possess authentic, synchronized GPS and sensor timestamps. The flight altitude was maintained between 40–60 m, corresponding to a ground spatial resolution of approximately 2.8 cm/pixel. The UAV followed predefined flight routes to perform grid-based inspections of the farmland area. During flight operations, an 80% forward overlap and a 70% side overlap were adopted to ensure continuous image coverage and reliable reconstruction quality. Inspection missions were carried out twice per week. Over the entire acquisition period, approximately 8700 raw UAV images were collected. After image quality screening, cropping, and redundancy removal, a total of 6420 valid inspection images were retained, structured appropriately for their distinct roles in the visual pre-training and multimodal alignment phases.

The annotation process followed a rigorous expert manual labeling protocol to ensure ground-truth reliability. Three agricultural experts with specialized knowledge in crop pathology and agronomy were recruited as annotators. Using the Computer Vision Annotation Tool (CVAT), they provided bounding box annotations for pest-induced damage and disease spots, and image-level categorical labels for growth assessment. To ensure consistency, an inter-annotator agreement check was conducted, achieving a Cohen’s Kappa coefficient of 0.86. The growth assessment task was categorized into three distinct physiological stages: Seedling Stage (Early), Reproductive Stage (Middle), and Maturing Stage (Late), reflecting the complete life cycle from emergence to harvest.

Regarding the class distribution, out of the 6420 total valid images, 3570 images represent healthy crop canopies, while the remaining 2850 images contain pest and disease symptoms, encompassing the 3760 annotated disease instances. Furthermore, our longitudinal data collection revealed a clear correlation between disease occurrence, seasonal microclimates, and crop growth levels. For instance, wheat powdery mildew predominantly emerged during the reproductive stage when crop canopy density and relative humidity were at their highest, whereas wheat stripe rust was more frequently observed transitioning from the late seedling to the early reproductive stages, heavily driven by specific seasonal temperature fluctuations.

Ground environmental information was continuously collected through agricultural Internet-of-Things sensor nodes deployed across the experimental farmland. A total of 18 monitoring nodes were arranged in a grid pattern across the experimental area. Regarding the scale mismatch between the 80 m sensor spacing and the centimeter-level imagery, we initially considered advanced spatial interpolation techniques such as Kriging or Inverse Distance Weighting. However, we ultimately implemented a nearest-neighbor spatial alignment strategy for two primary agronomic and mathematical reasons. First, the experimental farmland features an exceptionally flat topography and a highly homogeneous planting structure, meaning that the severe localized microclimatic gradients that typically occur in hilly or diverse terrains are greatly minimized here. Second, mathematically modeling a continuous spatial surface requires a sufficiently dense network of data points to generate an accurate semivariogram. Applying complex interpolation across 18 nodes over 48 hectares risks introducing unverified synthetic artifacts into the environmental data. Consequently, associating each UAV image with the exact, unaltered empirical data from the geographically closest sensor provides a more conservative and reliable environmental proxy than an interpolated guess. To address the temporal alignment between the 10 min interval sensor recordings and the continuous UAV flight imagery, we did not simply average the data around the image timestamps. Instead, we applied a linear temporal interpolation. For each exact image timestamp, we extracted the two closest sensor observations immediately before and after the image capture from the spatially assigned node to calculate the interpolated microclimatic value at the visual sampling time. Because the sensor network operates on a strict 10 min sampling interval, the maximum possible time difference between any given image capture timestamp and the nearest sensor record is mathematically bounded to exactly 5 min. Within such a brief 5 min window, macroscopic environmental variables such as ambient temperature and relative humidity remain highly stable, thereby ensuring that our temporal interpolation accurately reflects the true microclimatic conditions at the exact moment of visual capture without introducing temporal distortion. Furthermore, the model utilized a 24 h environmental time-series window prior to each flight to provide a microclimatic history for the captured phenotypic states. Each node was equipped with air temperature sensors, relative humidity sensors, and soil moisture sensors, and the collected data were transmitted through a LoRa wireless communication network. The sensor sampling interval was set to 10 min, resulting in approximately 98,500 environmental monitoring records throughout the entire observation period. Spatial location information was recorded using the UAV GPS module together with a farmland differential positioning system. By matching the image coordinates with farmland plot boundary vector data, spatial relationships among imagery, environmental data, and specific farmland plots were established. From an economic and commercial perspective, the multimodal dataset constructed in this study also provides value beyond benchmark evaluation. The spatiotemporally aligned UAV imagery, environmental sensor logs, and GPS records form a verifiable production record that can support agricultural e-commerce, third-party quality certification, and food safety supervision. Such structured records are consistent with certification requirements that involve production environments and pest or disease management evidence, and can therefore serve as reusable data assets for producers seeking access to premium market channels [59]. In addition, IoT- and blockchain-based traceability systems require continuous, georeferenced environmental and visual monitoring data similar to those captured in this dataset [60]. Thus, the released benchmark not only supports crop–environment interaction modeling but also provides a practical reference for commercially deployable agricultural data collection pipelines.

### 3.2. Data Preprocessing and Augmentation Strategy

In multimodal farmland inspection tasks, systematic data preprocessing and augmentation are essential to address inconsistencies in scale, noise interference, and temporal-spatial asynchrony across heterogeneous sources. For UAV imagery, we applied standard operations including bilinear interpolation for scale normalization to a fixed resolution, radial distortion correction based on the intrinsic calibration parameters of the Zenmuse P1 camera, and z-score normalization to mitigate illumination variations. For the ground environmental sensor data, preprocessing involved threshold-based anomaly detection, where outliers were explicitly defined as sensor readings deviating by more than three standard deviations from the local temporal mean, and these anomalous points were subsequently replaced using neighborhood averaging. Furthermore, the handling of missing values, which occasionally occurred due to temporary communication drops, was defined as applying local linear interpolation between the immediately adjacent valid sensor observations to ensure the continuity of the time-series data. Moving-average filtering was then applied to reduce high-frequency measurement noise. To achieve multimodal alignment, spatial mapping was performed using affine transformations to associate sensor geographic coordinates with the UAV image coordinate system. The use of an affine transformation is appropriate here because our experimental site in Wuyuan County is characterized by exceptionally flat topography, which minimizes perspective distortion caused by elevation changes. To mitigate potential errors introduced by UAV flight patterns, wind, or operator inconsistencies, we utilized the DJI Matrice 300 RTK drone, which provides centimeter-level positioning and high flight stability. It is important to clarify that this spatial mapping process was applied only to our field-captured images. Internet-sourced images were excluded from this coordinate transformation as they lack the necessary GPS and orientation metadata; instead, they contributed to the model through visual feature enrichment during pre-training. Furthermore, temporal alignment was achieved through a strictly localized linear interpolation. Because the UAV flights occurred twice a week while sensors recorded continuously every 10 min, the interpolation was exclusively performed between the two sensor readings immediately preceding and following each specific image capture timestamp. This highly localized alignment ensures that the assumption of a smooth, continuous signal remains perfectly valid for the brief 10 min window, allowing us to accurately estimate the microclimatic conditions at the precise moment of visual sampling. Finally, to enhance model generalization under complex agricultural conditions, we employed data augmentation strategies comprising random rotation, flipping, scale transformation, and brightness adjustment for the UAV images, alongside the injection of small Gaussian noise perturbations into the sensor time-series data to simulate environmental fluctuations. An additional preprocessing consideration with direct e-commerce relevance is data format standardization. To improve interoperability with downstream supply chain systems, including e-commerce traceability interfaces, certification databases, and customs electronic inspection platforms, the structured outputs of the proposed sensing framework are organized according to common agricultural traceability data specifications. Specifically, environmental sensor records are formatted with reference to ISO 22000 [62] food safety management requirements, while UAV imagery and disease annotations are structured to support traceability-oriented data exchange. Although this step does not change the data content, it reduces integration costs for potential deployment in agricultural digital supply chains. This is consistent with prior research showing that insufficient cross-platform data standardization remains a major barrier to digital quality certification in China’s agricultural e-commerce ecosystem.

### 3.3. Proposed Method

#### 3.3.1. Overall

Upon completing data preprocessing and alignment, heterogeneous inputs are organized into visual features, environmental sequences, and spatial location encodings for processing by the multimodal target perception network. Rather than a simple assembly of standard components, the proposed framework is designed as a synergetic architecture that models the intricate dependencies between visual phenotypic expressions and their corresponding microclimatic drivers. The framework integrates four core components: a visual perception branch, an environmental sensor branch, a location encoding module, and a multimodal fusion module, which are unified through a domain-specific cross-modal interaction logic. The operational logic of this integrated system is detailed in Algorithm 1.
**Algorithm 1** Workflow of the Multimodal Target Perception NetworkInput:UAV image *I*, Environmental sensor data *S*, GPS coordinates *P*Output:Pest detection results $Y_{1}$, Crop growth assessment $Y_{2}$1:Extract multi-scale visual features $F_{v}$ from *I* using convolutional extractors2:Embed *S* into environmental feature vectors $F_{s}$ via temporal encoding3:Map *P* into spatial representation $F_{p}$ using positional functions4:Perform cross-modal alignment: $F_{cm}=\mathrm{Align}\left(F_{v},F_{s},F_{p}\right)$ to bridge semantic gaps5:Apply multi-scale pyramid processing on $F_{cm}$ to handle scale variance6:Compute adaptive weights $w_{v}$, $w_{s}$, $w_{p}$ via dynamic gating mechanism7:Generate joint representation: $F_{\mathrm{joint}}=w_{v}F_{v}+w_{s}F_{s}+w_{p}F_{p}$8:Input $F_{\mathrm{joint}}$ into task-specific heads9:**return** $Y_{1}$ (Detection), $Y_{2}$ (Assessment)

Visual semantic features, including leaf structures and disease patterns, are extracted to produce multi-scale representations. Concurrently, environmental variables such as temperature and humidity are embedded into vectors characterizing microclimate conditions. To incorporate spatial context, geographic coordinates are mapped into high-dimensional representations within the location module. These features are subsequently processed by the cross-modal alignment module. The innovation here lies in the synergetic alignment process, which is essential for bridging the profound distribution discrepancies between high-dimensional spatial imagery and low-dimensional time-series sensor data by mapping them into a unified semantic space where environmental context can directly inform visual recognition.

The aligned features are then forwarded to the multi-scale target perception module. The justification for this module lies in the inherent scale variance of farmland targets, necessitating the simultaneous detection of microscopic disease spots and macroscopic canopy structures through a coordinated feature enhancement structure. Finally, a dynamic multimodal fusion and decision module is employed to address the fluctuating reliability of data sources under varying field conditions. Unlike fixed-weight combinations, this module utilizes a gating mechanism to adaptively weight multimodal features based on real-time data quality, generating a joint representation for final task-specific predictions. This collaborative architecture effectively transitions the system from simple visual recognition to a comprehensive, synergetic multi-source sensing framework specifically engineered for the complexities of precision agriculture.

#### 3.3.2. Cross-Modal Alignment and Collaborative Encoding Module

The core objective of the cross-modal alignment and collaborative encoding module is to map features from different modalities into a unified semantic representation space, thereby establishing structured associations between visual information and environmental sensing information.

**Figure 2:**
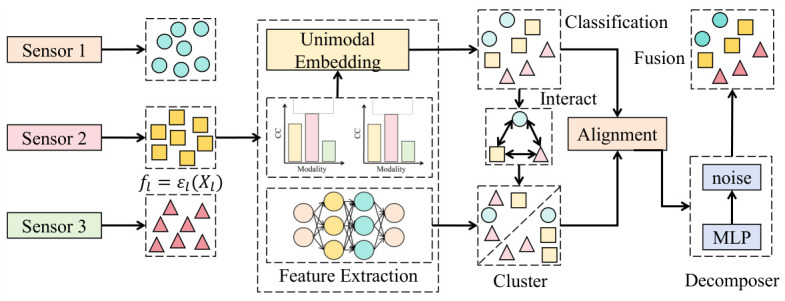
Schematic illustration of the cross-modal alignment and collaborative encoding module, showing the embedding, interaction, and fusion process of heterogeneous modal features in a unified semantic space.

As shown in Figure 2, the visual branch first produces visual representations extracted by the multi-scale feature extraction network, denoted as $F_{v}\in R^{N\times d}$, where *N* represents the number of spatial feature units extracted from the image and *d* represents the feature dimension. Meanwhile, the environmental sensor encoding branch maps multidimensional environmental variables such as temperature, air humidity, and soil moisture into vector representations denoted as $F_{s}\in R^{M\times d}$, where *M* represents the number of sensor variables. Location information is converted into spatial embedding vectors through a positional encoding function, represented as $F_{p}\in R^{1\times d}$. To achieve unified representation of cross-modal features, linear projection layers are first applied to map features from different modalities into a shared latent semantic space, which can be expressed as

(1) $$Z_{v}=W_{v}F_{v}+b_{v},\;Z_{s}=W_{s}F_{s}+b_{s},\;Z_{p}=W_{p}F_{p}+b_{p},$$

where $W_{v},W_{s},W_{p}\in R^{d\times d}$ denote projection matrices for each modality and $b_{v},b_{s},b_{p}$ denote bias terms. To further establish relationships between visual features and environmental information, a cross-modal attention mechanism is introduced. Through the query–key–value structure, feature interaction between modalities is modeled. In this structure, visual features act as query vectors, while sensor features act as key and value vectors. The attention weights are defined as

(2) $$A=\mathrm{Softmax}\left(\frac{Q_{v}K_{s}^{T}}{\sqrt{d}}\right),$$

where $Q_{v}=Z_{v}W_{q}$, $K_{s}=Z_{s}W_{k}$, and $V_{s}=Z_{s}W_{v}^{\prime }$, with $W_{q}$, $W_{k}$, and $W_{v}^{\prime }$ representing learnable parameter matrices. The cross-modal fused feature representation is expressed as

(3) $${\tilde{F}}_{v}=AV_{s}.$$

This mechanism enables visual features to be updated while incorporating environmental context, thereby allowing the model to capture potential correlations between environmental conditions and crop disease occurrence. To further enhance consistency across modalities, a cross-modal alignment loss is constructed in the feature space. By minimizing the distribution difference between modal representations, semantic alignment can be achieved. It is crucial to clarify that this alignment is not executed directly on the raw, highly heterogeneous data. Instead, prior to this step, both the high-dimensional visual inputs and the low-dimensional sensor sequences have been mapped into a shared, abstract latent semantic space through their respective modality-specific encoders and non-linear projection heads. Within this mathematically constructed hidden space, enforcing distribution similarity becomes a valid and necessary strategy. This constraint forces the network to capture the underlying semantic correlations between specific microclimatic conditions and their corresponding visual disease phenotypes, rather than attempting to match their disparate physical characteristics. This is expressed as

(4) $$L_{align}=∥\mu_{v}-\mu_{s}{∥}_{2}^{2}+{∥\Sigma_{v}-\Sigma_{s}∥}_{F}^{2},$$

where μ and Σ represent the mean and covariance matrices of the feature distributions from different modalities within the aforementioned shared latent semantic space. To further enhance feature representation capability, the cross-modal encoding module adopts a two-layer feed-forward network with hidden dimension d=256, combined with residual connections and layer normalization to ensure stable training of deep features. After processing by this module, visual, environmental, and spatial features are encoded into a unified semantic representation $F_{cm}$ and subsequently forwarded to the multi-scale target perception module for further analysis. Through this cross-modal alignment and collaborative encoding mechanism, instability caused by relying solely on visual information in complex farmland environments can be significantly alleviated. Environmental information thus becomes an important complementary source for visual perception, while the model’s understanding of crop disease occurrence conditions is enhanced, ultimately improving robustness and recognition accuracy in pest detection and crop growth assessment tasks. Beyond technical recognition performance, the semantically unified representations produced by the cross-modal alignment module also have commercial implications. By jointly encoding visual crop conditions and concurrent environmental context, these features can form structured quality descriptors for individual crop images. Such descriptors may provide objective, sensor-corroborated inputs for agricultural product grading in e-commerce supply chains, offering more informative and verifiable quality signals than unimodal visual classification. This is particularly relevant for premium markets, where traceable and environment-aware quality assurance is valued by consumers and institutional buyers [59], and where certified high-quality products can improve farm-level profitability through price premiums [9]. Therefore, the cross-modal alignment mechanism supports not only robust detection but also data-driven agricultural product quality certification.

It is highly important to distinguish our synergetic fusion approach from naive multimodal concatenation strategies. While standard concatenation directly merges heterogeneous sequences before applying transformer layers, it inherently fails to account for the profound distribution discrepancies and the fluctuating reliabilities between high-dimensional imagery and sparse sensor data in real-world agricultural environments. In contrast, our framework processes the modalities through a two-stage synergetic integration. First, it projects these modalities into a unified semantic space guided by a dedicated cross-modal alignment loss to bridge the heterogeneity gap. Subsequently, rather than passively transforming a concatenated sequence, we implement a dynamic multimodal gating mechanism. This module actively computes reliability weights for each modality based on real-time contextual conditions, ensuring that the final joint representation can dynamically suppress noisy signals and prioritize reliable priors, thereby achieving genuine adaptive multi-source sensing rather than simple feature aggregation.

#### 3.3.3. Multi-Scale Target Perception Module

The multi-scale target perception module is designed to simultaneously capture crop structural information and disease target features at different spatial scales within complex farmland environments. Since farmland imagery contains both large crop canopy regions and extremely small disease or pest spots, single-scale feature representations are insufficient for handling such scale variations. Therefore, a multi-scale feature enhancement structure is constructed based on the feature outputs of the visual backbone network. The backbone network generates hierarchical feature maps through successive convolution and downsampling operations, denoted as $\left\{C_{2},C_{3},C_{4},C_{5}\right\}$, where each layer corresponds to a different spatial resolution.

**Figure 3:**
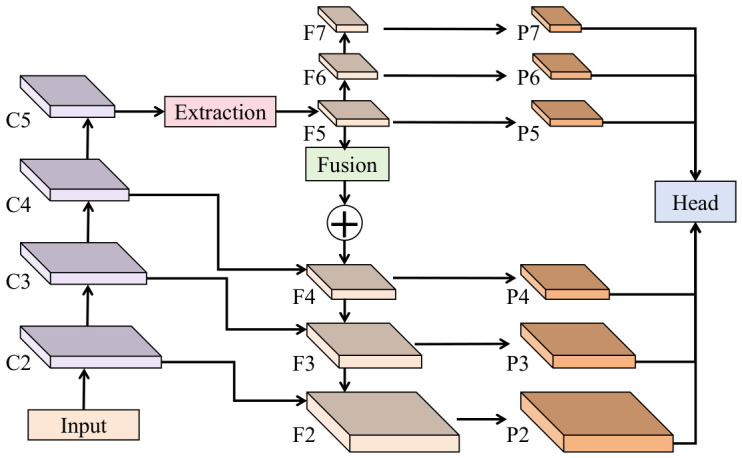
Architecture of the multi-scale target perception module, which integrates feature pyramid structures and deformable convolutions to jointly model and detect farmland targets at multiple spatial scales.

As shown in Figure 3, let the input image size be H×W. The feature map of the *i*-th layer of the backbone network can be represented as $C_{i}\in R^{\frac{H}{s_{i}}\times \frac{W}{s_{i}}\times d_{i}}$, where $s_{i}$ denotes the downsampling ratio and $d_{i}$ denotes the channel dimension. To enhance high-level semantic information while preserving low-level spatial details, the highest-level feature $C_{5}$ is first reconstructed through a multi-scale feature enhancement module to produce an intermediate representation $F_{5}$, which can be expressed as

(5) $$F_{5}=\phi \left({\mathrm{Conv}}_{3\times 3}\left(\sigma \left({\mathrm{Conv}}_{1\times 1}\left(C_{5}\right)\right)\right)\right),$$

where ${\mathrm{Conv}}_{1\times 1}$ performs channel compression and semantic projection, ${\mathrm{Conv}}_{3\times 3}$ captures local contextual information, σ(·) denotes a nonlinear activation function, and ϕ(·) represents normalization. A top-down feature propagation strategy is then employed to construct a multi-scale semantic pyramid, in which high-level features are progressively propagated to lower layers. For an arbitrary layer *k*, the fusion process can be expressed as

(6) $$F_{k}=\psi \left(\mathrm{Up}\left(F_{k+1}\right)⊕\Theta \left(C_{k}\right)\right),$$

where Up(·) denotes the upsampling operator, Θ(·) denotes channel alignment transformation of the original features, ⊕ represents element-wise fusion, and ψ(·) represents the combination of convolution and normalization operations. Through this process, multiple fused features $\left\{F_{2},F_{3},F_{4},F_{5}\right\}$ are obtained. After obtaining multi-level fused features, deformable convolution is introduced to dynamically adjust spatial sampling locations, thereby improving the perception capability for irregular disease regions. The response of deformable convolution can be expressed as

(7) $$y\left(p\right)=\sum_{q\in \Omega }w\left(q\right)\cdot x\left(p+q+\Delta q\right),$$

where *p* denotes the output feature location, Ω denotes the convolution sampling set, w(q) denotes convolution weights, and Δq denotes learnable offsets. This structure enables sampling locations to adaptively change according to target shapes, thereby improving recognition of irregular disease textures. To further construct multi-scale detection structures, additional feature layers $\left\{F_{6},F_{7}\right\}$ are generated through progressive downsampling of fused features, expressed as

(8) $$F_{k+1}=\eta \left({\mathrm{Conv}}_{3\times 3}\left(F_{k}\right)\right),$$

where η(·) denotes a convolution operation with stride-based downsampling. Ultimately, a complete multi-scale feature set $\left\{P_{2},P_{3},P_{4},P_{5},P_{6},P_{7}\right\}$ is obtained, where each feature map corresponds to target perception at a specific scale and is connected to detection heads for prediction. For an arbitrary feature map $P_{i}$, the prediction function can be expressed as

(9) $${\hat{y}}_{i}=g\left(P_{i};\theta_{i}\right),$$

where g(·) denotes the detection prediction function and $\theta_{i}$ denotes the parameters of that layer. From a theoretical perspective, multi-scale feature representation can be interpreted as approximating the target function under different spatial resolutions. By constructing a set of hierarchical feature mappings, more comprehensive target representations can be obtained. When the feature space satisfies

(10) $$F=\overset{7}{\underset{i=2}{⋃}}P_{i},$$

targets can be represented simultaneously across multiple scale spaces, thereby improving overall perception capability. In farmland inspection scenarios, this structure effectively addresses the large variation in crop disease spot sizes. Low-level feature layers enhance detection of early-stage disease spots and pest regions, while high-level semantic features are more effective for identifying large-scale crop growth regions and disease propagation patterns. In addition, deformable convolution enables the model to adapt to complex leaf texture variations, thereby improving detection robustness for irregular disease structures and providing more stable multi-scale perception performance in complex agricultural environments. The early detection capability enabled by the multi-scale target perception module also provides economic value for agricultural supply chain management. By identifying pest and disease symptoms at early morphological stages, the proposed module allows farm operators and e-commerce procurement teams to adjust sourcing plans, initiate precision interventions, and update inventory expectations before large-scale crop quality degradation occurs. This is particularly important for agricultural e-commerce fulfillment, where production shortfalls may lead to financial losses and reputational risks. Early detection can also support more targeted pesticide application, reducing residue risks and improving compliance with high-value export market requirements. Since cross-border agricultural e-commerce is sensitive to food safety compliance failures and quality rejection events [61], the multi-scale detection structure contributes not only to agronomic improvement but also to supply chain reliability and regulatory compliance.

#### 3.3.4. Dynamic Multimodal Fusion and Decision Module

After the multi-scale target perception module, the model has obtained visual semantic representations from multiple spatial scales, while also preserving environmental sensor features and spatial location information produced by the cross-modal alignment module. To achieve adaptive multimodal information fusion, a dynamic multimodal fusion and decision module is constructed. Through sequential interaction encoding and modality-aware weight learning mechanisms, joint modeling of multi-source information is achieved.

**Figure 4:**
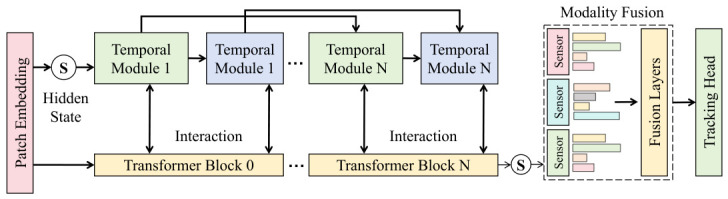
Architecture of the dynamic multimodal fusion and decision module, which performs temporal interaction modeling and modality-aware routing to achieve adaptive fusion of multi-source information and final prediction.

As shown in Figure 4, this module first maps feature representations from the visual feature pyramid, environmental sensor embeddings, and positional encodings into a unified feature dimension, generating a modality feature sequence through linear embedding layers. Let the multi-scale visual feature set be denoted as $V=\{V_{1},V_{2},\ldots ,V_{n}\}$, the environmental sensor representation be denoted as *S*, and the spatial position encoding be denoted as *P*. All modal features are reorganized into a unified sequence $Z=\{z_{1},z_{2},\ldots ,z_{m}\}$ and projected through an embedding mapping function expressed as

(11) $$z_{i}=\psi \left(W_{e}x_{i}+b_{e}\right),$$

where $x_{i}$ denotes input features from different modalities, $W_{e}$ denotes the embedding projection matrix, $b_{e}$ denotes the bias term, and ψ(·) denotes a nonlinear activation function. After embedding, multimodal temporal interaction modeling is conducted using a multi-layer Transformer architecture. Each Transformer layer contains multi-head self-attention operations and feed-forward networks, enabling dependency learning among different modalities. Let the input feature matrix be *Z*. The self-attention computation can be expressed as

(12) $$H=\mathrm{Softmax}\left(\frac{\left(ZW_{q}\right){\left(ZW_{k}\right)}^{T}}{\sqrt{d}}\right)\left(ZW_{v}\right),$$

where $W_{q}$, $W_{k}$, and $W_{v}$ are learnable parameter matrices and *d* denotes the feature dimension. After several layers of interaction encoding, a shared semantic representation across modalities is formed, enabling environmental information to dynamically modulate visual representations. To further achieve adaptive multimodal fusion, a modality-aware routing network is introduced after the Transformer output. This network learns the importance weights of each modality through a gating function. Let the fused feature set be $\left\{h_{1},h_{2},\ldots ,h_{m}\right\}$. The modality weights can be expressed as

(13) $$\alpha_{i}=\frac{\exp(g\left(h_{i}\right))}{\sum_{j=1}^{m}\exp\left(g\left(h_{j}\right)\right)},$$

where g(·) denotes the mapping function of the gating network. The final fused representation can be written as

(14) $$F=\sum_{i=1}^{m}\alpha_{i}h_{i}.$$

To analyze the stability of the fusion strategy, it can be interpreted from the perspective of information preservation. Specifically, by interpreting the normalized dynamic gating weights as the probability distribution of modality selection, the probability density function of the fused feature space can be mathematically modeled as a mixture distribution of the individual modality feature spaces. Let the information entropy of each modality feature distribution be $H_{i}$, and the entropy of the fused representation be H(F). When the weights satisfy the normalization constraint $\sum \alpha_{i}=1$, the concavity property of Shannon entropy guarantees that the entropy of a mixture distribution is bounded below by the linear combination of the individual entropies. Consequently, the fused representation satisfies

(15) $$H\left(F\right)\geq \sum_{i=1}^{m}\alpha_{i}H_{i}.$$

Therefore, from an information-theoretic perspective, the dynamic fusion strategy preserves multimodal information to the greatest extent and prevents the loss of information from any single modality. The fused representation is then fed into the final decision head for pest recognition and crop growth prediction. The prediction function can be expressed as

(16) $$y=\sigma (W_{d}F+b_{d}),$$

where $W_{d}$ denotes the weight matrix of the decision layer, $b_{d}$ denotes the bias term, and σ(·) denotes a nonlinear function. Through this dynamic multimodal fusion and decision structure, the model can automatically adjust the importance of visual information and sensor information according to environmental conditions. When visual observations are affected by illumination variations or occlusion, environmental sensor features provide complementary information, thereby improving model robustness in complex agricultural environments. In addition, cross-modal interaction encoding enables the model to learn potential relationships between crop growth conditions and environmental variables, resulting in more stable and accurate predictions in pest detection and crop growth assessment tasks.

## 4. Results and Discussion

### 4.1. Experimental Configuration

#### 4.1.1. Hardware and Software Platform

In terms of the hardware platform, the experiments were conducted on a high-performance deep learning computing server. The server was equipped with an Intel Xeon series multi-core processor to provide stable data preprocessing and task scheduling capability. In addition, an NVIDIA RTX 3090 GPU was employed for deep neural network training and inference computation, with a memory capacity of 24 GB, which was sufficient to support the training requirements of high-resolution UAV imagery and multimodal feature fusion models. The system memory was 128 GB DDR4, which enabled large-scale data loading and parallel computation. A high-speed NVMe solid-state drive was also utilized to improve data reading efficiency and reduce the impact of I/O bottlenecks during the training process. With the support of this hardware configuration, model training and experimental evaluation could be executed efficiently and reliably, thereby providing a stable computational environment for the construction and optimization of the multimodal farmland inspection perception network.

Regarding the software platform, the experimental environment was built on the Ubuntu 20.04 operating system. The deep learning framework was implemented using PyTorch (version 1.12.1) for network construction and model training, while CUDA 11.x and cuDNN were utilized to accelerate GPU-based parallel computation. Data processing and scientific computing were primarily conducted within the Python (version 3.8.10) ecosystem. Specifically, NumPy (version 1.21.6) was used for numerical computation, OpenCV (version 4.6.0) was applied for image processing and augmentation operations, Pandas was used for sensor data processing and time-series analysis, and Matplotlib (version 3.5.2) was employed for visualization of experimental results. During model training, the automatic differentiation mechanism provided by PyTorch was used for gradient computation, while GPU parallelization was adopted to accelerate model optimization. These configurations ensured that the entire experimental pipeline maintained good stability and reproducibility.

In terms of hyperparameter configuration, the constructed dataset was first divided into training, validation, and testing subsets with a ratio of 7:2:1. This specific partition ratio was selected based on empirical deep learning practices tailored to our medium-sized dataset of 6420 images. Allocating 70% of the data for training ensures that the multimodal network is exposed to sufficient phenotypic and microclimatic diversity to learn robust feature representations. The 20% validation set is large enough to provide a statistically reliable estimate of model performance during training, which is crucial for accurately triggering the learning rate decay strategy and preventing overfitting. The remaining 10%, comprising approximately 640 images, serves as a completely unseen hold-out test set, providing a rigorous and objective evaluation of the final generalization capability without excessively reducing the data available for model optimization. To ensure a fair and rigorous comparison, all models in this study, including the proposed framework and various baseline models, were trained using a unified input resolution of 640×640 pixels. Regarding model initialization, the visual backbones utilized weights pretrained on the MS COCO dataset to leverage transfer learning and accelerate convergence, while all newly proposed components, such as the environmental sensor branch and the multimodal fusion module, were initialized randomly and trained from scratch on our specific farmland dataset. During model training, the Adam optimization algorithm was adopted. The initial learning rate was set to α=0.001, and the weight decay coefficient was set to $1\times 10^{-4}$ to reduce overfitting. The batch size was set to 16 to balance computational efficiency and GPU memory consumption. The maximum number of training iterations was set to 100 epochs for all comparative experiments to maintain a consistent training schedule. During training, a learning rate decay strategy was applied. When the validation loss no longer decreased for several consecutive epochs, the learning rate was automatically reduced to improve convergence stability. In addition, to further enhance the reliability and generalization capability of the experimental evaluation, a 5-fold cross-validation strategy was employed. Specifically, the dataset was divided into five subsets of approximately equal size. In each round of experiments, one subset was used as the test set, while the remaining subsets were used for model training and validation. The average performance across multiple training runs was then adopted as the final evaluation result, thereby reducing the randomness introduced by dataset partitioning and improving the reliability of the experimental findings.

#### 4.1.2. Baseline Models and Evaluation Metrics

In the experiments, several representative object detection methods and multimodal fusion approaches were selected as baseline models for comparison. These included Faster R-CNN [63], YOLOv5 [64], RetinaNet [65], Mask R-CNN [66], UAV + Sensor Concatenation [67], and Cross-Modal Transformer [68]. Faster R-CNN is a classical two-stage object detection framework in which candidate regions are generated by a region proposal network and subsequently refined through classification and bounding box regression. The detection process can be formulated as predicting bounding box parameters (x,y,w,h) and category probability p(c). This approach is characterized by relatively high detection accuracy and stable performance in complex scenarios. YOLOv5 is a single-stage object detection model that directly performs object localization and classification through an end-to-end convolutional network. The primary advantage of this method lies in its high computational efficiency and fast inference speed, which makes it suitable for real-time inspection scenarios. RetinaNet is an improved single-stage detection framework that introduces the focal loss function to address the issue of class imbalance. This approach demonstrates improved detection capability for difficult samples and small targets. Mask R-CNN extends Faster R-CNN by incorporating an additional pixel-level segmentation branch on top of the detection framework, enabling the extraction of precise object contours. This design allows both object location and detailed shape information to be obtained simultaneously. UAV + Sensor Concatenation represents a basic multimodal fusion strategy in which UAV image features $F_{v}$ and sensor features $F_{s}$ are directly concatenated to form a joint feature representation $F=[F_{v},F_{s}]$ that is subsequently fed into a prediction network. The advantage of this approach lies in its simplicity and low implementation cost while enabling preliminary utilization of multimodal information. Cross-Modal Transformer is a multimodal fusion model based on the Transformer architecture. It employs attention mechanisms to enable information interaction between different modalities. This approach is capable of capturing global dependencies between multimodal features and thereby improving cross-modal fusion effectiveness.

Multiple commonly used object detection evaluation metrics were employed to comprehensively assess model performance, including Precision, Recall, mAP, and F1-score. Precision measures the proportion of correctly predicted positive samples among all predicted positive samples, while Recall measures the proportion of true positive samples correctly detected by the model. mAP evaluates the overall detection performance across different threshold conditions, and F1-score reflects the balance between precision and recall. The mathematical definitions of these evaluation metrics are given as follows:

(17) $$Precision=\frac{TP}{TP+FP},$$

(18) $$Recall=\frac{TP}{TP+FN},$$

(19) $$F1=2\times \frac{Precision\times Recall}{Precision+Recall},$$

(20) $$AP=\int_{0}^{1}P\left(R\right)dR,$$

(21) $$mAP=\frac{1}{N}\sum_{i=1}^{N}AP_{i}.$$

In these expressions, TP denotes the number of true positives, representing correctly detected objects. FP denotes the number of false positives, representing background regions incorrectly detected as targets. FN denotes the number of false negatives, representing existing targets that were not detected. Precision indicates the accuracy of detection results, while Recall reflects the completeness of detection results. AP represents the average precision of a single category across different recall levels, which is computed from the area under the Precision–Recall curve. mAP represents the mean average precision across all categories, where *N* denotes the total number of target categories and $AP_{i}$ represents the average precision of the *i*-th category.

### 4.2. Farmland Pest and Disease Detection Task

This experiment was designed to systematically evaluate the performance differences between various visual detection models and multimodal fusion models on the farmland pest and disease detection task, thereby validating the effectiveness and advantages of the proposed multimodal target perception network in complex agricultural scenarios. Specifically, several representative object detection models were selected as comparison baselines, including typical two-stage detection models, single-stage detection models, and preliminary multimodal fusion approaches. Performance comparisons were conducted under a unified dataset and consistent evaluation metrics in order to analyze the adaptability of different model structures to farmland inspection tasks.

**Figure 5:**
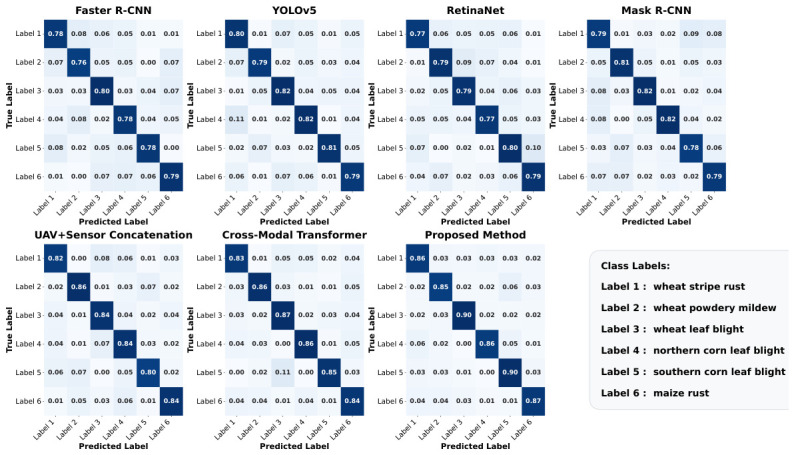
Confusion matrices of different models on the farmland pest detection task, illustrating classification performance across six disease categories.

As shown in Table 2 and Figure 5, conventional visual detection methods such as Faster R-CNN, RetinaNet, and Mask R-CNN achieved reasonable performance on the pest and disease detection task, although their overall effectiveness remained constrained by the complexity of farmland scenes. For example, as a typical two-stage detection framework, Faster R-CNN achieved relatively good detection accuracy through the region proposal mechanism. However, because candidate region generation relies on fixed visual feature representations, its detection stability remained limited under illumination variation and background interference. As a single-stage detection model, YOLOv5 improved detection efficiency through an end-to-end prediction mechanism and achieved a relatively balanced performance in terms of Precision and Recall. Nevertheless, its feature representation primarily relied on single visual information, which still led to certain difficulties in recognizing small-scale disease spots and complex textured environments. RetinaNet improved category imbalance through the introduction of focal loss and therefore outperformed several conventional models in detection performance. However, environmental contextual information could still not be fully exploited under complex agricultural backgrounds.

In contrast, models incorporating multimodal information exhibited more substantial performance gains. The UAV + Sensor Concatenation method improved overall performance over purely visual methods to some extent by directly concatenating UAV imagery with environmental sensor data, thereby enhancing the model’s perception of environmental variation. However, because this method lacked an effective cross-modal semantic alignment mechanism, information collaboration between modalities remained limited. The Cross-Modal Transformer further improved all evaluation metrics by enabling cross-modal feature interaction through the attention mechanism, allowing the model to learn associations between visual information and environmental information within a unified feature space. The proposed method achieved the best performance in terms of Precision, Recall, F1, and mAP, which can be mainly attributed to the integration of multimodal collaborative modeling and multi-scale target perception mechanisms. From the perspective of model structure, the cross-modal alignment module established associations between visual features and environmental variables in the feature space, enabling auxiliary judgment based on temperature, humidity, and other environmental conditions during disease spot recognition, thereby improving detection stability. Meanwhile, the multi-scale target perception module simultaneously modeled crop canopy structures and small-scale disease spot features under different spatial resolutions, allowing strong recognition capability to be maintained under complex farmland backgrounds. In addition, the dynamic multimodal fusion mechanism adaptively adjusted feature weights according to the reliability of different data modalities, thereby reducing the influence of noise from any single modality on detection results. These structural advantages enabled the model to achieve more stable feature representation capability in complex agricultural environments and therefore obtain superior overall performance on the farmland pest and disease detection task. Translating the performance improvements in Table 2 into economic terms further highlights the practical value of the proposed method. Its precision advantage over the strongest visual-only baseline, Swin-Transformer, indicates a reduced risk that symptomatic crop material remains undetected and enters post-harvest handling or commercial channels without remediation. Given that global crop losses caused by pests and diseases are estimated to reach approximately 21.5% in wheat and 22.6% in maize [8], reliable early detection can support targeted intervention, reduce yield losses, and improve product quality consistency. For agricultural e-commerce operators, where quality consistency and certification status strongly influence platform visibility and consumer willingness to pay [59], reducing undetected pest and disease risks can generate direct commercial benefits. Therefore, the improved detection accuracy of the proposed method represents both an agronomic advance and an economic asset for e-commerce-integrated agricultural production systems.

### 4.3. Crop Growth Assessment Task

This experiment was designed to systematically evaluate the performance differences among different types of models on the crop growth assessment task, thereby verifying the importance of multimodal information in the recognition of farmland growth states. Compared with the pest and disease detection task, crop growth assessment depends not only on the visual morphological characteristics of plants but also on changes in environmental conditions. Therefore, several representative models were selected as comparative baselines, including typical convolutional neural network models, self-attention-based visual models, temporal models using only environmental sensor data, and multimodal fusion models. Under a unified dataset and evaluation protocol, the adaptability of different model structures to agricultural scenarios was analyzed by comparing their performance in terms of Precision, Recall, F1, and Accuracy.

**Figure 6:**
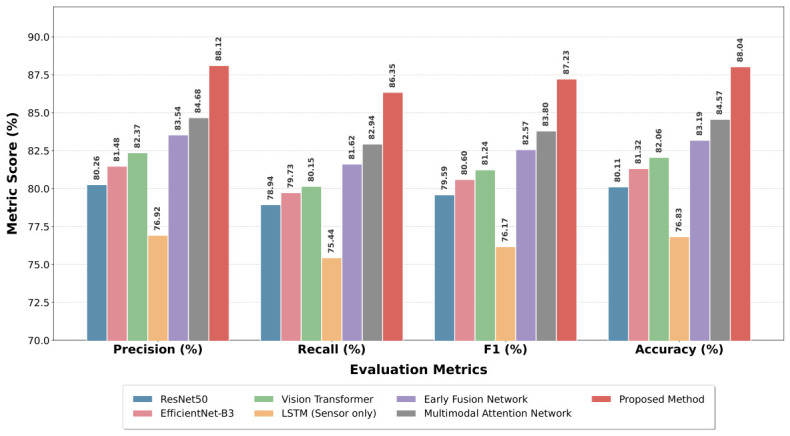
Bar chart comparing the performance of different models on the crop growth assessment task, showing their results in terms of Precision, Recall, F1, and Accuracy.

As shown in Table 3 and Figure 6, conventional visual models such as ResNet50 extracted relatively stable image features and therefore achieved baseline performance across all metrics. However, because such models primarily rely on local convolutional structures, their capability to understand the global structural state of crop growth remained limited. EfficientNet-B3 improved feature representation efficiency through a more optimized network design and scale reuse mechanism, and therefore slightly outperformed ResNet50 in metrics such as Precision and Accuracy. Vision Transformer established global dependencies among different image regions through the self-attention mechanism, allowing crop canopy structures and spatial distribution patterns to be captured more effectively, which resulted in further overall performance improvement. On the other hand, the LSTM model using only environmental sensor data achieved relatively lower overall performance, indicating that a single environmental variable stream is insufficient to comprehensively describe crop growth variation. Although sensor data can reflect growth conditions such as temperature, humidity, and soil moisture, direct morphological information of plants is absent, which limits the model when distinguishing different growth levels. The Early Fusion Network significantly outperformed unimodal methods by integrating visual features and sensor data at an early stage, enabling both information sources to be jointly utilized. The Multimodal Attention Network further improved all metrics by weighting features from different modalities through the attention mechanism, thereby learning correlations between visual information and environmental conditions. In comparison, the proposed method achieved the best performance in terms of Precision, Recall, F1, and Accuracy. This superiority can be mainly attributed to the simultaneous introduction of cross-modal alignment, multi-scale visual modeling, and dynamic fusion mechanisms. From the perspective of mathematical characteristics, the cross-modal alignment mechanism established associations between visual features and environmental variables within a unified feature space, enabling crop growth assessment to be performed not only on the basis of image textures but also in conjunction with environmental conditions. At the same time, multi-scale feature modeling captured both crop leaf details and overall canopy structures, thereby improving the representation capability for different growth stages. The dynamic fusion strategy reduced the influence of noise from any single modality through adaptive weight adjustment, allowing stable prediction performance to be maintained in complex agricultural environments. Therefore, more accurate and robust recognition results were achieved by the proposed method in the crop growth assessment task. The economic implications of improved crop growth assessment accuracy extend to agricultural supply chain planning and e-commerce inventory management. Accurate real-time recognition of crop growth stages can support harvest timing forecasts, just-in-time cold chain scheduling, and reduced perishability-related losses in fresh produce logistics. This is consistent with studies on China’s agricultural e-commerce supply chains, where timely crop status information is identified as important for resilience and operational efficiency [57]. Moreover, the three-stage output of the proposed model, including Seedling, Reproductive, and Maturing stages, corresponds to key commercial decision windows, supporting pre-harvest procurement planning and fulfillment scheduling. Continuous monitoring records can also improve producers’ access to premium buyer channels and price negotiation capacity through documented crop growth and quality history [9]. Therefore, the crop growth assessment function provides market-relevant quality signals for e-commerce-oriented agricultural operations.

### 4.4. Ablation Study on Detection Task

The purpose of this ablation experiment was to analyze the specific contributions of key modules in the proposed multimodal target perception network to performance improvement on the farmland pest and disease detection task, thereby validating the rationality and effectiveness of the model design. The experiment was conducted by progressively removing different functional modules while keeping other components unchanged. Comparative analysis was performed for the cross-modal alignment and collaborative encoding module, the multi-scale target perception module, and the dynamic multimodal fusion module. In addition, a model using only the visual branch was employed as the basic reference.

**Figure 7:**
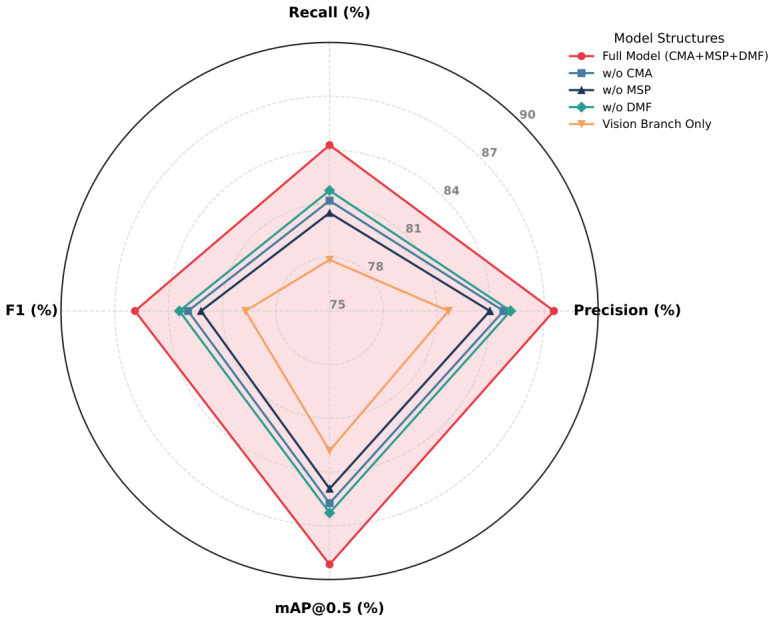
Radar chart comparing multiple performance metrics of different model structures in the farmland pest detection task.

As shown in Table 4 and Figure 7, the full model achieved the best performance in terms of Precision, Recall, F1, and mAP, indicating that the collaborative operation of multiple modules significantly enhanced detection capability in complex agricultural scenarios. The ablation results reveal a hierarchical contribution pattern among the modules. While the Visual branch only configuration serves as the baseline, the introduction of the multi-scale target perception (MSP) module yields the most significant quantitative improvement, as evidenced by the 4.23% drop in mAP when it is removed. This confirms that robust visual modeling remains the primary driver for identifying small-scale phenotypic symptoms. However, the multimodal components, namely cross-modal alignment (CMA) and dynamic multimodal fusion (DMF), provide a critical secondary layer of performance enhancement by bridging the gap between pure visual recognition and contextual sensing.

When only the visual branch was retained, overall model performance decreased substantially. This result indicates that single visual information is insufficient for stable disease target recognition in complex farmland environments, because disease spots are often characterized by small scales, indistinct textures, and strong background interference. Crucially, the combined contribution of CMA and DMF accounts for approximately 2–4% of the mAP gain. Although this is numerically secondary to the MSP module, these multimodal modules act as contextual stabilizers. The CMA module ensures that environmental priors can directly inform the feature extraction process, while the DMF module provides a reliability gating mechanism.

From the perspective of model structure, the MSP module exerted the most pronounced influence on overall performance, as the largest decrease in mAP was observed after its removal. This finding indicates that scale variation is highly important for model recognition capability in the farmland pest and disease detection task. Disease spots in images are often small and unevenly distributed. While MSP handles the geometric and structural complexities of the targets, the multimodal components address the environmental uncertainty. Performance also declined after the removal of the dynamic multimodal fusion module; although its quantitative contribution appears more modest compared to other modules, its primary role is to serve as a reliability guard. By adaptively weighting modalities, it prevents the model from over-relying on visual signals when illumination or background conditions are sub-optimal. The cross-modal alignment module established associations between visual information and environmental variables, which improved detection stability by providing biological constraints for disease occurrence. Overall, the three modules formed a complementary structure where the visual modeling provides the core content and the multimodality provides the necessary context, thereby achieving more accurate results in complex farmland environments.

To further elucidate the adaptive mechanism of the dynamic multimodal fusion module, we conducted a qualitative analysis of the modality weights under varying environmental conditions. Specifically, we examined the dynamic gating weights assigned to the visual, environmental, and spatial modalities for identical crop regions captured under contrasting circumstances. Under optimal visual conditions, characterized by clear illumination and absent occlusion, the fusion module predominantly prioritized the visual modality, assigning it an average attention weight of approximately 0.75, while the environmental and spatial modalities received weights of 0.15 and 0.10, respectively. This distribution indicates that when phenotypic features are pristine, the network logically relies heavily on direct morphological evidence. Conversely, we analyzed challenging scenarios where the crop canopy was severely obscured by heavy shadows from adjacent structures or dense weed blockages. In these instances of degraded visual quality, the gating mechanism dynamically adjusted its focus, reducing the visual modality weight to approximately 0.40 and correspondingly increasing the environmental sensor weight to 0.45, with the spatial encoding weight rising slightly to 0.15. This explicit redistribution of attention demonstrates that the proposed dynamic multimodal fusion module does not passively aggregate features but actively compensates for visual uncertainty by amplifying its reliance on localized microclimatic priors, thereby maintaining robust detection performance even when direct visual observation is heavily compromised.

### 4.5. Ablation Study on Assessment Task

The main purpose of this ablation experiment was to analyze the specific roles of the key modules in the proposed multimodal target perception network for the crop growth assessment task and to verify the contributions of different modules to the overall model performance. By progressively removing the cross-modal alignment and collaborative encoding module, the multi-scale target perception module, and the dynamic multimodal fusion module from the full model, while setting a visual-branch-only model as the reference, the influence of each module on predictive capability could be observed more clearly.

**Figure 8:**
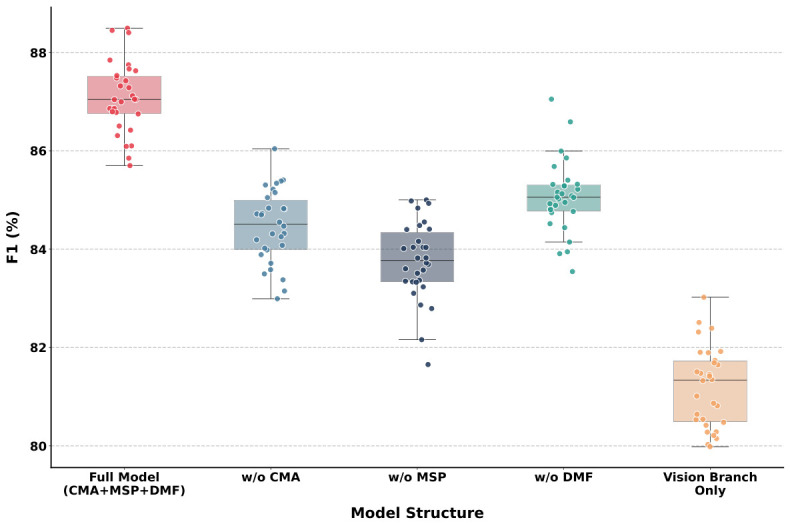
Comparison of F1-score distributions for different model structures in the ablation study, illustrating the contribution of each module to overall performance improvement.

As shown in Table 5 and Figure 8, the full model achieved the highest performance in terms of Precision, Recall, F1, and Accuracy, indicating that the multimodal collaborative structure effectively improved the accuracy and stability of crop growth assessment. The ablation results reveal a highly balanced synergy between the visual enhancements and the multimodal integration. Specifically, while the multi-scale target perception module accounts for approximately half of the total accuracy gain compared to the pure visual baseline, the combined multimodal components encompassing cross-modal alignment and dynamic multimodal fusion contribute the remaining half. This distribution clearly illustrates the exact role of each modality within the framework. When only the visual branch was used, overall model performance decreased substantially, indicating that single visual information is insufficient to fully characterize the true growth state of crops. Crop growth status is reflected not only in changes in leaf color and morphology but is also closely related to environmental conditions such as temperature, humidity, and soil moisture. Therefore, while visual feature extraction acts as the baseline for recognizing plant structures, multimodality serves as the foundational pillar for system robustness. In the absence of environmental constraints, the visual model becomes highly vulnerable to illumination variation and background interference in complex farmland scenes, which sharply reduces recognition stability.

From the perspective of individual module contribution, a clear performance decline was observed after removal of the cross-modal alignment module, indicating that semantic associations between different modal features are highly important for the crop growth assessment task. This module established mapping relationships between visual features and environmental variables, allowing the model to combine environmental variation information with crop canopy structures and leaf texture analysis, thereby producing more stable feature representations. A further decline in performance was observed after removal of the multi-scale target perception module, confirming its primary role in handling spatial scale differences, including both local leaf structural variations and overall canopy density changes. Without multi-scale feature representation capability, part of the critical structural information is easily lost during feature representation. The removal of the dynamic multimodal fusion module also led to performance degradation, because the information quality of different modalities varies across time and environmental conditions, and the adaptive fusion mechanism dynamically adjusts the importance of each modality to reduce the influence of noisy information on predictions. It is critical to note that in real-world precision agriculture, the substantial accuracy margin provided by these multimodal components often dictates the operational threshold of the system. This specific margin prevents system failure under adverse weather conditions, transitioning the model from a controlled-environment algorithm to a field-ready application. Overall, these three modules formed a complementary structure that enabled comprehensive analysis of crop growth states from the perspectives of visual structure, environmental conditions, and multi-scale spatial information, thereby yielding more stable and accurate prediction results for the crop growth assessment task.

### 4.6. Discussion

In practical agricultural production environments, farmland inspection is not only a technical issue but is also directly related to production efficiency and farmers’ economic returns. The proposed approach demonstrates significant advantages over traditional agricultural management modes, where crop growth and pest conditions are assessed through manual field inspection. While manual methods may be workable for small-scale plots, their efficiency and accuracy are insufficient for large-scale intensive production. Our multimodal framework enables systematic monitoring of extensive areas within a short time through the collaborative sensing of UAV imagery and ground sensor data. The primary technical advantage of this framework is its enhanced robustness against environmental noise, such as illumination variations and occlusions, which typically degrade the performance of vision-only systems. From the perspective of agricultural economics, early pest and disease detection through this system can significantly reduce marginal control costs, as the investment required for localized treatment at an early stage is far lower than that required for large-scale control after disease diffusion. Therefore, such technology contributes to improving the input–output efficiency and the overall quality of agricultural products.

Furthermore, the real-world applicability of this multimodal perception system is evident in its support for precision crop growth management. Farmland conditions are often affected by soil fertility differences and local microclimate variations, leading to substantial growth heterogeneity. Traditional coarse management often results in resource waste, such as over-irrigation or excessive fertilization. Our method provides a precise decision-making basis by identifying the potential causes of growth variation through joint environmental data analysis. This supports differentiated management strategies, such as localized irrigation for areas with moisture deficits, thereby reducing input costs while maintaining yield. This transformation from experience-driven to data-driven decision-making improves the transparency of production information and enhances resource allocation efficiency in regional agricultural systems.

Implications for Agricultural E-commerce Value Chain Integration. The monitoring outputs generated by the proposed framework, including time-stamped and GPS-anchored pest detection records, environmental sensor histories, and growth stage classifications, constitute a structured quality evidence archive for agricultural traceability systems. Blockchain-based traceability architectures increasingly require continuous multi-source field data as verifiable evidence inputs for consumer trust and regulatory compliance [60]. Therefore, the proposed framework supports a dual-use deployment model, in which the same infrastructure used for agronomic monitoring can also generate documentation for quality certification and premium market access. This commercial value is supported by agricultural e-commerce studies showing that traceable products with verifiable production histories can command price premiums, with trust in the traceability system playing a key role [59]. Empirical evidence from China further indicates that combining product quality certification with digital sales channels generates higher revenue than either strategy alone, especially for high-value crops [9]. Thus, the economic return of the proposed framework should be evaluated not only through yield improvement and input cost reduction, as supported by precision agriculture evidence [7], but also through the market premiums and supply chain access enabled by its traceability-oriented data outputs.

E-commerce-Oriented Deployment Models for Smallholder Farmers. A major barrier to precision agriculture adoption is the high cost faced by small- and medium-scale farms, which often cannot independently finance UAV inspection, IoT sensor networks, and data management infrastructure [69]. Existing studies show that large farms benefit from economies of scale, whereas farms under 100 hectares often face higher per-unit deployment costs that may offset expected productivity gains [7]. In agricultural e-commerce contexts, a feasible solution is a cooperative deployment model, where platforms or agricultural cooperatives provide shared sensing infrastructure and recover costs through service fees or revenue-sharing mechanisms linked to quality premiums. This model is supported by China’s rural digital economy practices. For example, the Tudouec platform in Inner Mongolia showed that platform-mediated supply chain integration can reduce intermediary costs, improve quality consistency, and create new income pathways for smallholders [58]. Guo et al. also reported that platform-based agricultural supply chain integration enhances resilience and profitability for smaller operators with limited market access and logistics capacity [57]. Applied to the proposed framework, cooperative deployment would allow smallholders to access standardized pest monitoring, growth assessment, and quality certification data at shared infrastructure costs, while platform operators benefit from improved product quality, lower return risks, and stronger consumer trust.

However, the practical implementation of the proposed approach also faces certain limitations that must be addressed for broader adoption. A significant constraint is the requirement for initial hardware investment and the maintenance of a coordinated UAV-IoT network, which may present a barrier for small-scale farmers or regions with limited technical infrastructure. Regarding the potential concern of spatial overfitting in the Location Encoding module, we wish to clarify that while absolute GPS coordinates were utilized in our experimental demonstration, the framework architecture is fundamentally designed to be coordinate-agnostic. In cross-regional applications, the system can utilize normalized local coordinates or relative spatial offsets, allowing the model to focus on the universal biological clustering patterns of pest and disease spread rather than specific geographic values. This design choice ensures that the learned spatial–temporal priors remain transferable across diverse cultivation regions. Additionally, the performance of the multimodal model is sensitive to the quality of sensor data, which can exhibit abnormal fluctuations under extreme weather conditions. By acknowledging these constraints, we emphasize that while the proposed framework offers a robust technical pathway for precision agriculture, its large-scale deployment should be accompanied by cost-effective hardware solutions and adaptive model calibration strategies to ensure high generalization performance across different regional contexts.

### 4.7. Limitations

Although the proposed multimodal farmland intelligent inspection method achieved promising experimental results in the pest and disease detection task and the crop growth assessment task, several limitations still remain. First, the current model was mainly constructed and validated on a specific region and a limited range of crop types. Although stable performance was achieved within the experimental plots, its adaptability under larger spatial scales, broader climate conditions, and different cultivation systems still requires further validation. Significant differences in disease occurrence mechanisms, soil conditions, and management practices across regions may influence the generalization performance of the model. Second, multimodal data acquisition relies on the coordinated deployment of UAV inspection systems and ground sensor networks. In practical applications, such deployment requires a certain level of hardware investment and maintenance cost, which may still present barriers for small- and medium-scale farmers. In addition, environmental sensor data may exhibit abnormal fluctuations or missing values under complex climatic conditions, which places higher demands on model stability. These limitations indicate that the applicability of the proposed framework may still be affected by regional differences, deployment conditions, and data quality in real agricultural production environments.

From the perspectives of agricultural economics and e-commerce integration, several limitations should be acknowledged. First, although the monitoring outputs are formatted to support common agricultural traceability specifications, the absence of unified cross-platform data standards in China’s agricultural e-commerce ecosystem means that practical deployment still requires platform-specific integration, increasing implementation costs [62]. Second, the economic value of certification and traceability data depends on regional institutional conditions, including certification infrastructure, farmer digital literacy, and target-market regulations, which may vary across regions and crop types [69]. Third, the present study does not empirically evaluate the return on investment of the proposed framework under real deployment conditions; future work should conduct cost–benefit analyses covering hardware investment, data management, platform integration, productivity gains, and market access benefits [6]. Finally, large-scale agricultural monitoring also raises data governance concerns, including farm-level data ownership, farmer privacy, and the commercial use of production records, which may affect participation in e-commerce traceability systems [62].

## 5. Conclusions

With the development of smart and digital agriculture, farmland monitoring is shifting from manual inspection toward intelligent sensing and data-driven decision-making. To address the limited robustness of single-vision methods in complex agricultural environments and the insufficient use of environmental information, this study proposes a multimodal target perception network for intelligent farmland inspection. The proposed framework integrates UAV imagery, ground environmental sensor data, and spatial location information to jointly support pest and disease detection and crop growth assessment. By introducing cross-modal alignment, multi-scale target perception, and dynamic multimodal fusion, the model can collaboratively represent heterogeneous sensing information in a unified feature space and improve perception robustness under complex field conditions. Experimental results on the constructed multimodal farmland dataset demonstrate the effectiveness of the proposed method. For the pest and disease detection task, the model achieved 87.53% Precision, 84.27% Recall, 85.87% F1-score, and 89.16% mAP, showing better detection accuracy than representative visual detectors and multimodal baselines. For the crop growth assessment task, the model achieved 88.12% Precision, 86.35% Recall, 87.23% F1-score, and 88.04% Accuracy, indicating that the integration of environmental and spatial information can effectively enhance crop status recognition. The ablation results further confirm the contribution of each key module: cross-modal alignment improves the consistency of heterogeneous features, multi-scale target perception enhances the detection of farmland targets with different sizes, and dynamic fusion adaptively balances the contributions of visual, environmental, and spatial modalities. Overall, this study provides an effective multimodal perception framework for AI-driven farmland monitoring. The findings suggest that collaborative modeling of UAV imagery, environmental sensor data, and spatial information can improve the accuracy and robustness of agricultural sensing systems, thereby supporting precision agricultural decision-making and early risk warning. In future work, we will further expand the dataset across different crop types, growth stages, regions, and seasons to improve model generalization. We also plan to investigate lightweight deployment strategies for UAV and edge-computing platforms, strengthen temporal modeling of long-term sensor data, and explore more interpretable multimodal fusion mechanisms to support practical field applications. In addition, future work will explore integration with agricultural e-commerce supply chain management systems by connecting real-time field monitoring outputs with inventory planning, quality certification, and consumer-facing traceability interfaces. Blockchain-based protocols will be investigated to securely link multimodal monitoring records with product-level digital identities, enabling verifiable provenance tracking from sensor to consumer [60]. Cost-effective deployment architectures for smallholder farming will also be studied based on cooperative platform models in agricultural e-commerce [57,58]. Finally, empirical economic evaluations will be conducted to assess market access improvement, quality premium realization, and supply chain loss reduction, thereby complementing the technical metrics and supporting investment decisions by farm operators, cooperatives, and e-commerce platforms [6,7].

## Figures and Tables

**Table 1:** Statistics of the multimodal farmland inspection dataset.

Data Type	Source	Characteristics	Quantity
UAV images	Multirotor UAV RGB camera	Resolution ≈2.8 cm/pixel	6420 images
Sensor data	Temperature, humidity, moisture sensors	Sampling interval: 10 min	98,500 records
GPS location data	UAV GPS and positioning system	Image spatial coordinates	6420 records
Disease annotations	Expert manual labeling	Diseases	3760 instances
Crop growth status labels	Expert agronomic evaluation	Three-level growth status	6420 samples

**Table 2:** Performance comparison of different methods on the farmland pest and disease detection task.

Method	Precision (%)	Recall (%)	F1 (%)	mAP@0.5 (%)	mAP@0.5:0.95 (%)	GFLOPs	Processing Time (ms)
Faster R-CNN	78.42	74.85	76.59	79.31	48.52	180.5	85.0
YOLOv5	81.37	77.94	79.62	82.48	52.14	45.3	22.5
RetinaNet	79.65	75.28	77.40	80.17	49.33	145.2	65.4
Mask R-CNN	80.14	76.83	78.45	81.26	50.87	210.8	110.2
YOLOv10	85.12	82.45	83.76	87.03	57.82	50.1	20.8
Swin-Transformer	85.84	81.92	83.83	87.51	58.45	240.4	95.6
UAV + Sensor Concatenation	83.06	79.21	81.09	84.37	54.21	55.2	28.3
Cross-Modal Transformer	84.11	80.36	82.19	85.42	55.68	85.6	45.7
Proposed method	87.53	84.27	85.87	89.16	60.34	75.4	38.5

**Table 3:** Performance comparison of different methods on the crop growth assessment task.

Method	Precision (%)	Recall (%)	F1 (%)	Accuracy (%)
ResNet50	80.26	78.94	79.59	80.11
EfficientNet-B3	81.48	79.73	80.60	81.32
Vision Transformer	82.37	80.15	81.24	82.06
LSTM (Sensor only)	76.92	75.44	76.17	76.83
Early Fusion Network	83.54	81.62	82.57	83.19
Multimodal Attention Network	84.68	82.94	83.80	84.57
Proposed method	88.12	86.35	87.23	88.04

**Table 4:** Ablation study on the farmland pest and disease detection task.

Model Structure	Precision (%)	Recall (%)	F1 (%)	mAP@0.5 (%)
Full model (CMA + MSP + DMF)	87.53	84.27	85.87	89.16
w/o CMA	84.72	81.16	82.90	85.74
w/o MSP	83.95	80.48	82.18	84.93
w/o DMF	85.11	81.73	83.39	86.28
Visual branch only	81.64	77.86	79.70	82.81

**Table 5:** Ablation study on the crop growth assessment task.

Model Structure	Precision (%)	Recall (%)	F1 (%)	Accuracy (%)
Full model (CMA + MSP + DMF)	88.12	86.35	87.23	88.04
w/o CMA	85.74	83.41	84.56	85.28
w/o MSP	84.91	82.63	83.75	84.67
w/o DMF	86.17	84.02	85.08	86.03
Visual branch only	82.36	80.21	81.27	81.94

## Data Availability

The data and source code supporting the findings of this study are currently maintained in a private repository for the peer-review process. To ensure full scientific reproducibility and to facilitate further development within the research community, both the complete multimodal farmland inspection dataset and the source code for our proposed synergetic sensing framework will be made publicly available at https://github.com/Aurelius-04/MSF4UAV.git (accessed on 20 May 2026) immediately upon the acceptance of this manuscript.
